# IL-38 Alleviates Inflammation in Sepsis in Mice by Inhibiting Macrophage Apoptosis and Activation of the NLRP3 Inflammasome

**DOI:** 10.1155/2021/6370911

**Published:** 2021-12-17

**Authors:** Yun Ge, Juan Chen, Yanting Hu, Xinyi Chen, Man Huang

**Affiliations:** Department of General Intensive Care Unit, The Second Affiliated Hospital of Zhejiang University School of Medicine, Hangzhou 310052, China

## Abstract

Interleukin- (IL-) 38 is an emerging cytokine with multiple functions involved in infection and immunity. However, the potential role of IL-38 in the host immune response during sepsis remains elusive. Herein, we investigated if macrophages in septic mice express IL-38, the molecular mechanisms behind its expression, and the downstream effects of its expression. In mouse peritoneal macrophages, lipopolysaccharide (LPS) upregulated IL-38 and its receptor IL-36R, and the resulting IL-38 shifted macrophages from a M1 to M2 phenotype. Moreover, exposure to IL-38 alone was sufficient to inhibit macrophage apoptosis and LPS-driven activation of the NOD-, LRR-, and pyrin domain-containing 3 (NLRP3) inflammasome. These effects were partly abrogated by IL-38 downregulation. In septic mice, IL-38 markedly lowered serum concentrations of proinflammatory cytokines and greatly improved survival. Conversely, IL-38 blockade aggravated their mortality. Collectively, these findings present IL-38 as a potent immune modulator that restrains the inflammatory response by suppressing macrophage apoptosis and activation of the NLRP3 inflammasome. IL-38 may help protect organs from sepsis-related injury.

## 1. Introduction

Despite the progress with antimicrobial agents and modern medical techniques, sepsis remains a devastating syndrome and the major cause of mortality among intensive care unit patients [1]. Sepsis is a severe condition in which the host immune response fails to resolve infection, resulting in a sustained systemic inflammatory response and multiple organ dysfunction [2]. Despite the development of novel drug candidates [3, 4], the majority of clinical trials for sepsis treatment have failed, and therapeutic options for sepsis remain limited. Therefore, further studies are needed into novel potential therapeutic targets for treating sepsis.

Cytokines are crucial players in the host immune response, so an in-depth understanding of cytokine-mediated immune responses during sepsis may provide hope for novel immune regulatory therapies [5]. Recently, we reviewed the substantial progress in understanding how interleukin- (IL-) 1 family of cytokines contributes to the development of sepsis [6]. These cytokines act as endogenous innate immune signals during the host response. Among them, IL-37, IL-33, IL-18, IL-1*β*, and IL-1*α* have been demonstrated to exert pleiotropic effects on various immune cells during sepsis [7–11]. The work from our laboratory [12] and others [13] has shown that IL-36 can greatly enhance the host immune response and improve prognosis of septic mice.

In inflammatory and autoimmune disorders, various types of immune cells, including Th1, Th17, and regulatory T cells, overexpress IL-38, a member of the IL-1 family [14]. In fact, IL-38 may be useful as a clinical biomarker for predicting sepsis development [15, 16]. IL-38 is widely expressed in various tissues, including the brain, heart, spleen, placenta, and tonsils. IL-38 tightly binds to several receptors, including IL-1 receptor accessory protein-like 1, IL-1 receptor 1, and the IL-36 receptor (IL-36R). It exerts anti-inflammatory activities by blocking several proinflammatory molecules and signaling pathways, such as those involving nuclear factor-*κ*B and mitogen-activated protein kinases [15–19]. In bone marrow-derived dendritic cells challenged with lipopolysaccharide (LPS), IL-38 downregulates the proinflammatory mediator IL-6 [18]. We also found that IL-38 could improve the immune functions of regulatory T cells and inhibit the expansion of effector T cells following LPS stimulation [20]. In addition, early treatment with IL-38 reduced the mortality of septic mice [20]. These studies suggest that IL-38 can control detrimental inflammatory cascades, including in sepsis. However, exactly how IL-38 influences sepsis and its outcomes remains to be clarified.

In this study, we aimed to examine IL-38 expression in macrophages and the downstream effects of that expression in a mouse model of sepsis.

## 2. Materials and Methods

### 2.1. Animals and Sepsis Model

C57BL/6J mice (six-week-old, male, weight 20-25 g) were provided by the Experimental Animal Center, Zhejiang University, Hangzhou, China (SCXK2014-0004). All experiments and procedures were performed in accordance with the Scientific Investigation Board of the Second Affiliated Hospital of Zhejiang University School of Medicine, Hangzhou, China.

The sepsis model was established by cecal ligation and puncture- (CLP-) induced polymicrobial sepsis as previously described [12, 20]. In brief, mice were anesthetized with 0.3% sodium pentobarbital. The cecum was exposed, ligatured at its external third, and punctured with a 22-gauge needle. The cecum was then returned to the peritoneal cavity, and the incisions were closed. All mice received (subcutaneous administration) 1 mL of saline for resuscitation immediately after surgery. Sham-operated mice served as a control. The survival of mice was observed and recorded twice daily for 7 days.

### 2.2. *In Vivo* Administration and Blockade of IL-38

Mice were injected intraperitoneally with recombinant mouse IL-38 (1 *μ*g per mouse, Adipogen, San Diego, CA) 2 h before CLP. For *in vivo* blockade of IL-38, rat polyclonal anti-mouse IL-38 antibody (50 *μ*g per mouse; R&D Systems, Minneapolis, MN, USA) was injected intraperitoneally immediately after CLP, and a booster dose (50 *μ*g) was injected 24 h later. As a control, phosphate-buffered saline (PBS) was administered in a similar fashion.

### 2.3. Isolation of Macrophages and *In Vitro* Treatments

Primary peritoneal macrophages were obtained from mice according to a previous study [21] under aseptic conditions. The cells were incubated in RPMI 1640 medium (Solarbio, Beijing, China) supplemented with 10% fetal bovine serum. The purity of the isolated macrophages was determined before each experiment by flow cytometry using a F4/80 PE-Cyanine 7 antibody (eBioscience, San Diego, CA, USA). Only macrophage preparations with a purity of >90% were used.

Cells were counted using a hemocytometer (Shanghai RuiYu Biotech Co. Ltd., China) by trypan blue exclusion assay. Macrophages were treated with different concentrations of LPS (0.25, 0.5, 1 *μ*g/mL; Sigma-Aldrich, St. Louis, MO, USA) for 12, 24, or 48 h. Cells were treated with recombinant mouse IL-38 at 50, 100, or 200 ng/mL for 24 h.

### 2.4. siRNA Transfection

Macrophages were transfected with small interference RNA (siRNA) against IL-38 or a nontargeting siRNA as control following the manufacturer's instructions. Transfected cells were incubated for 48 h in medium and then used for *in vitro* experiments. The efficiency of knockdown was tested by Western blotting for IL-38 expression.

### 2.5. Phagocytosis Assay

Macrophages (2 × 10^5^) were cultured with neutral red dye (Solarbio, Beijing, China) for 30 min at 37°C, washed with PBS (Solarbio), and observed using a light microscope (Leica, Mannheim, Germany), followed by the OD540 test using a microtiter plate reader (Tecan).

### 2.6. Bacteria-Killing Assay

To measure the ability of macrophages to kill bacteria, macrophages (1 × 10^5^) were incubated with murine L1210 leukemia cells (2 × 10^4^; ATCC) at 37°C for 24 h, and the OD450 test was then carried out using a microtiter plate reader (Tecan).

### 2.7. Quantitative Reverse Transcription-PCR (qRT-PCR)

After macrophages were treated as described above, total mRNA was extracted from 4 × 10^6^ cells using Trizol (Invitrogen, Carlsbad, CA, USA) following the manufacturer's instructions. The mRNA was reverse-transcribed using an iScript™ kit (BioRad, Hercules, CA, USA), and the mRNA concentration was estimated based on absorbance at 260 and 280 nm in the Nanodrop system. The mRNA levels of target genes were quantified using the SYBR Green Master Mix (Qiagen, Hilden, Germany) on a CFX96™ Real-Time PCR Detection System (BioRad). Amplification efficiency was 0.90–0.99. All data were normalized to *β*-actin mRNA levels.

### 2.8. Western Blot

Cells (4 × 10^6^) were lysed on ice with RIPA lysis buffer (Beyotime, China). Protein amount was quantified using the bicinchoninic acid (BCA) method (Beyotime) as indicated by the manufacturer. The protein samples were separated by sodium dodecyl sulfate polyacrylamide gel electrophoresis (SDS-PAGE) and transferred to polyvinylidene fluoride (PVDF) membranes. After blockade with QuickBlock Blocking Buffer (Beyotime), the PVDF membranes were incubated at 4°C overnight with the following antibodies at a concentration of 1 : 1000 in PBS-Tween : polyclonal rabbit antibodies against NLPR3 (Cell Signaling Technology Inc., Beverly, MA, USA.), adapter protein apoptosis-associated speck-like protein (ASC) (Cell Signaling Technology Inc.), IL-1*β* (Cell Signaling Technology Inc.), cleaved-caspase 1 (Cell Signaling Technology Inc.), Bax (Cell Signaling Technology Inc.), Bcl-2 (Cell Signaling Technology Inc.), cleaved-caspase 3 (Cell Signaling Technology Inc.), or IL-36R (Abcam, Cambridge, England); polyclonal rat antibody against mouse IL-38 (R&D Systems, Minneapolis, MI.); or polyclonal mouse antibody against *β*-actin (Santa Cruz Biotechnology, Santa Cruz, CA). Membranes were incubated with secondary antibodies at a concentration of 1 : 5000 for 2 h at room temperature. The specific bands were visualized by enhanced chemiluminescence (Beyotime) and ECL detection system (BioRad, Hercules, CA, USA).

### 2.9. Flow Cytometry

Cultured macrophages (5 × 10^5^) were incubated at 4°C for 30 min with anti-mouse antibodies against CD206 PE (eBioscience), mouse CD11b APC (eBioscience), or mouse F4/80 PE-Cyanine 7 (eBioscience). For apoptosis analysis, macrophages (5 × 10^5^) were examined using 7-AAD and Annexin V-PE (BD, San Diego, CA.) at 4°C for 30 min. Flow cytometry was performed in a FACSCalibur (BD Bioscience San Jose, CA, USA).

### 2.10. Laser Scanning Confocal Microscopy

Cultured cells were fixed using 4% paraformaldehyde for 20 min. Cells were washed with PBS for three times and were preblocked with 1% bovine serum albumin for 30 min. Then, cells were stained with anti-IL-38 or anti-IL36R antibodies and 4′,6-diamidino-2-phenylindole (DAPI) (Solarbio, Beijing, China). Confocal microscopy was performed using a laser scanning confocal microscope (Leica, Mannheim, Germany) according to the manufacturer's recommendations.

### 2.11. Serum Biochemistry and Cytokine Measurements

Serum of mice was obtained from peripheral blood extracted 24 h after CLP. Levels of activity or concentration of the following analytes were determined using ELISA kits following the manufacturer's instructions: aspartate aminotransferase (AST), creatinine, and alanine aminotransferase (ALT) (Sigma-Aldrich), as well as IL-38 (R&D Systems), IL-10 (ExCell Biology, Shanghai, China), TNF-*α* (ExCell Biology), IL-6 (ExCell Biology), and IL-1*β* (ExCell Biology). Plates were analyzed using a microplate reader (Tecan, SPARK 10 M, Austria).

### 2.12. Statistical Analyses

All analyses were performed using SPSS 20.0 software (IBM, Chicago, IL, USA) and a statistical significance definition of *P* < 0.05. All data are shown as the mean ± standard deviation of at least three independent experiments. Numbers of samples are indicated in the figure legends. Comparisons between groups were tested using the Brown-Forsythe test or, when appropriate, one-way ANOVA followed by Dunnett's test. In mouse survival studies, Kaplan-Meier curves were generated and compared using the log rank test.

## 3. Results

### 3.1. Mouse Macrophages Increase Expression of IL-38 and Its Receptor IL-36R upon LPS Stimulation

**Figure 1:**
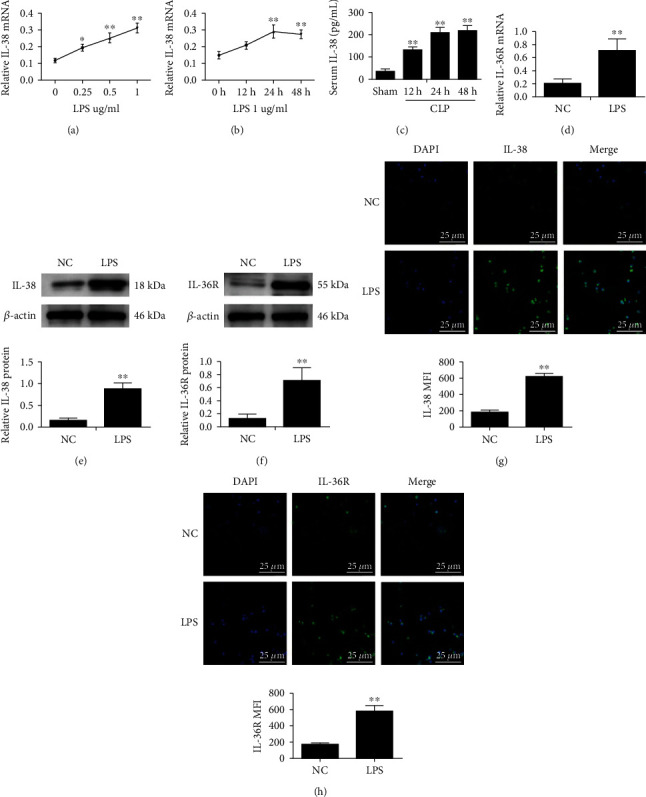
Expression of IL-38 and its receptor Il-36R in mouse macrophages treated with LPS. (a) IL-38 normalized mRNA expression in mouse macrophages stimulated with LPS at the indicated concentrations for 12, 24, and 48 h. *β*-Actin served as the housekeeping gene. (b) IL-38 normalized mRNA expression in mouse macrophages stimulated with 1 *μ*g/ml LPS for 12, 24, or 48 h. *β*-Actin was used as the housekeeping gene. *n* = 3; ^∗^*P* < 0.05 and^∗∗^*P* < 0.01 vs. the normal control (NC) group. NC was PBS treatment. (c) Serum concentrations of IL-38 in mice at the indicated timepoints after cecal ligation and puncture (CLP), based on ELISAs. *n* = 6; ^∗∗^*P* < 0.01*vs.* the sham group; (d) IL-36R normalized mRNA expression in mouse macrophages stimulated with LPS (1 *μ*g/ml; 24 h). *β*-Actin was used as housekeeping gene. (e, f) Representative western blot image (left panels) and quantification (right panels) of IL-38 and IL-36R protein levels of macrophages stimulated with 1 *μ*g/mL LPS for 24 h. *β*-Actin protein levels were used for normalization. (g, h) Representative immunofluorescence images of IL-38 (green) and IL-36R (green) of macrophages treated as in panels (d, e). Nuclei were stained with DAPI (blue). Magnification, ×600. Scale bar, 25 *μ*m. ^∗∗^*P* < 0.01*vs*. the NC group. Results are shown for three independent experiments.

We previously found that IL-38 is expressed in mouse CD4^+^CD25^+^ regulatory T cells and is upregulated upon exposure to inflammatory stimuli [19]. Here, we explored the expression of IL-38 and its receptor IL-36R in mouse macrophages under inflammatory conditions. We isolated peritoneal mouse macrophages and treated them with various concentrations of LPS (0.25, 0.5, and 1 *μ*g/mL) for 12, 24, or 48 h. LPS induced expression of IL-38 and IL-36R (Figures 1(a), 1(a), and 1(d)–1(f)). The highest induction of IL-38 occurred after the addition of 1 *μ*g/mL LPS for 24 h. IL-38 concentration was also dramatically elevated in the serum of septic mice (Figure 1(c)). Moreover, immunofluorescence experiments showed that IL-38 and IL-36R localized primarily to the cytoplasm (Figures 1(g) and 1(h)).

### 3.2. IL-38 Polarizes Macrophages toward M2 Anti-inflammatory Profile under LPS Stimulation

**Figure 2:**
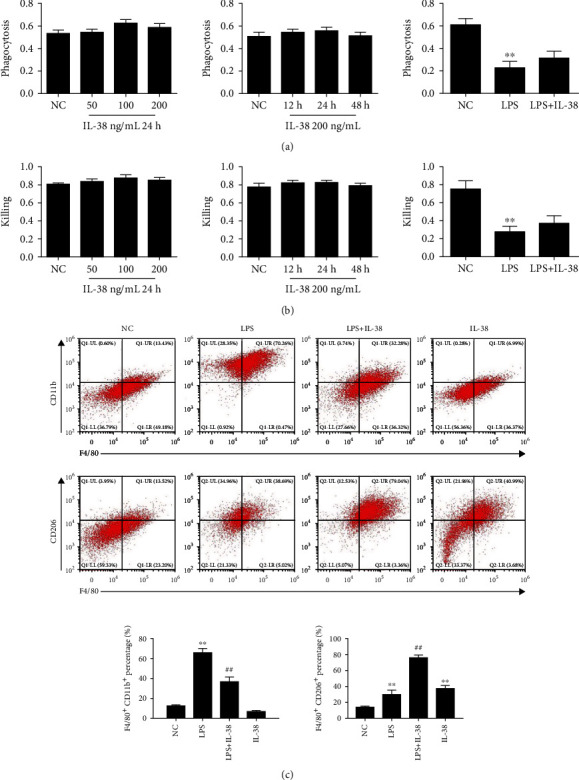
Effects of IL-38 on macrophage functions under LPS stimulation. (a) Phagocytic activity of peritoneal mouse macrophages upon LPS stimulation (1 *μ*g/mL) and treatment with IL-38 at the indicated concentrations and time. Phagocytic activity was measured using the OD540 test. Phagocytic activity of macrophages treated with LPS (1 *μ*g/mL) and IL-38 (200 ng/mL) for 24 h in the right panel. Shown are arbitrary numbers. (b) Killing of L1210 cells by macrophages treated as indicated. Shown are arbitrary numbers. Killing activity of macrophages treated with LPS (1 *μ*g/mL) and IL-38 (200 ng/mL) for 24 h in the right panel. ^∗^*P* < 0.05 and ^∗∗^*P* < 0.01, *vs*. the NC group. NC was PBS treatment. *n* = 6 independent experiments. (c) Representative flow cytometry plots of macrophages treated for 24 h with PBS or LPS (1 *μ*g/mL) alone or with recombinant IL-38 (200 ng/mL). The M1 phenotype was F4/80^+^ CD11b^+^, and the M2 phenotype was F4/80^+^ CD206^+^. Quantification of the experiment shown in the panel. The normal control (NC) group was PBS treatment. ^∗^*P* < 0.05 and ^∗∗^*P* < 0.01*vs*. the NC group. *^##^P* < 0.01*vs*. the LPS group. *n* = 6 independent experiments.

Macrophages can modify their phenotype in response to the microenvironment, particularly the presence of certain cytokines [19, 21]. The cells can adopt a proinflammatory M1 state or an anti-inflammatory M2 state. We treated mouse macrophage cultures with LPS and recombinant mouse IL-38 in order to better detect phenotypic effects of IL-38. Our data suggested that IL-38 had little effect on phagocytosis or killing abilities of macrophages (Figures 2(a) and 2(b)). After LPS stimulation, M1-type macrophage populations (F4/80^+^/CD11b^+^) markedly increased while M2-polarized macrophages (F4/80^+^/CD206^+^) were low (Figure 2(c)). Conversely, IL-38 increased the M2 population while decreasing the M1 population, compared with the LPS-alone group (Figure 2(c)).

### 3.3. IL-38 Inhibits the Ability of LPS to Activate the NLRP3 Inflammasome in Macrophages

**Figure 3:**
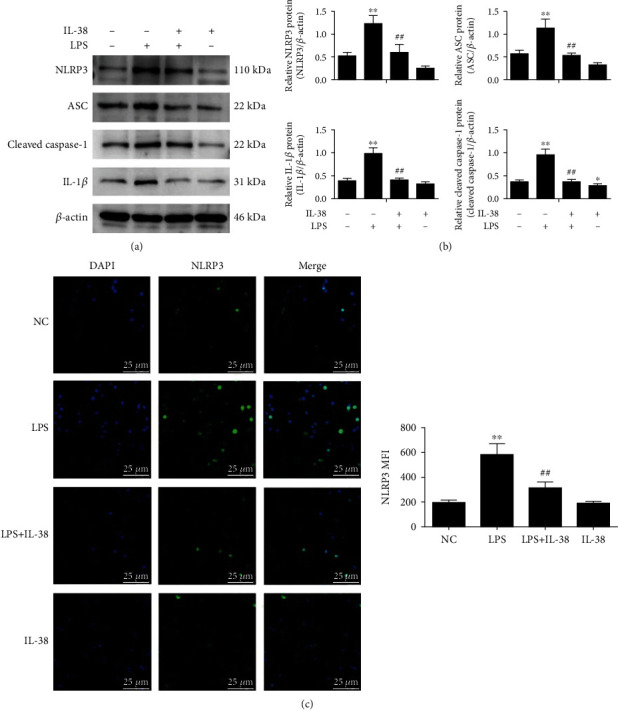
IL-38 dampens LPS-induced activation of the NLRP3 inflammasome in macrophages. (a) Representative western blot image of indicated proteins in macrophages stimulated with LPS (1 *μ*g/mL; 24 h) and/or IL-38 (200 ng/mL; 24 h). *β*-Actin protein was used for normalization. (b) Quantification of three independent experiments as shown in panel. (c) Representative immunofluorescence images of NLRP3 (green) of macrophages treated as in (a). Nuclei were stained with DAPI (blue). Magnification, ×600. Scale bar = 25 *μ*m. ^∗^*P* < 0.05 and ^∗∗^*P* < 0.01*vs*. the normal control (NC) group. NC was PBS treatment. *^##^P* < 0.01*vs*. the LPS group. *n* = 3 independent experiments.

Based on our observation that LPS and IL-38 can influence the inflammatory status of macrophages, we next explored the role of both factors on the activation of the NLRP3 inflammasome. NLRP3 inflammasome includes NLRP3 protein, ASC, and caspase 1. Once activated, interactions among these proteins convert the cytokine precursors (i.e., pro-IL-1*β*) into biologically active forms and initiate inflammatory responses [22]. LPS upregulated IL-1*β*, cleaved-caspase 1, ASC, and NLRP3 in macrophages (Figure 3). Conversely, cotreatment with IL-38 and LPS downregulated NLRP3 inflammasome proteins, compared to LPS alone (Figure 3). In fact, IL-38 returned NLRP3 proteins to similar levels as in control cells not treated with LPS.

### 3.4. IL-38 Inhibits the Ability of LPS to Induce Apoptosis in Macrophages

**Figure 4:**
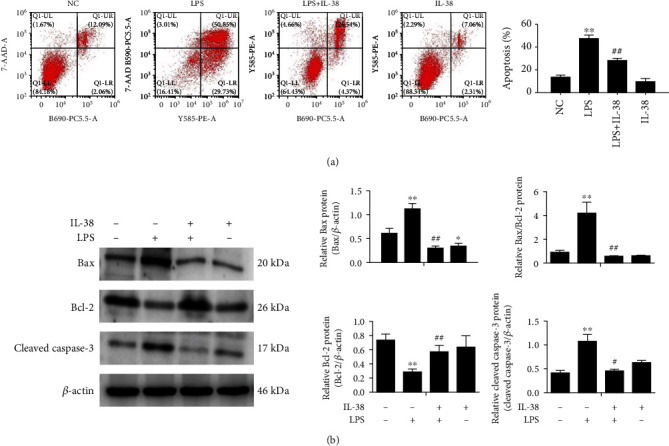
IL-38 inhibits LPS-induced apoptosis of macrophages. (a) Representative flow cytometry plots of macrophages treated with PBS or LPS (1 *μ*g/mL; 24 h) alone or with recombinant IL-38 (200 ng/mL; 24 h) in order to evaluate apoptosis (left panel). Percentages of apoptotic (Annexin V+) macrophages (right panel). (b) Representative western blot image (left panels) and quantification (right panels) of the indicated proteins in macrophages stimulated with LPS (1 *μ*g/mL; 24 h) and/or IL-38 (200 ng/ml; 24 h). *β*-Actin protein was used for normalization. Normal control (NC) group was PBS treatment. ^∗^*P* < 0.05 and ^∗∗^*P* < 0.01, vs. the NC group; ^#^*P* < 0 05 and ^##^*P* < 0.01, vs. the LPS group. *n* = 3 independent experiments.

During sepsis, the number of apoptotic macrophages increases, suggesting that such apoptosis helps drive the condition [21]. Indeed, in our macrophage cultures, LPS clearly enhanced the rate of apoptosis as well as upregulated Bax and cleaved-caspase 3 proteins, while downregulating Bcl-2 expression (Figure 4). As a result, the ratio of Bax to Bcl-2 increased. These effects were partially reversed by cotreatment with IL-38 and LPS together.

### 3.5. IL-38 Knockdown Impacts Polarization, Inflammasome Activation, and Apoptosis in Macrophages

**Figure 5:**
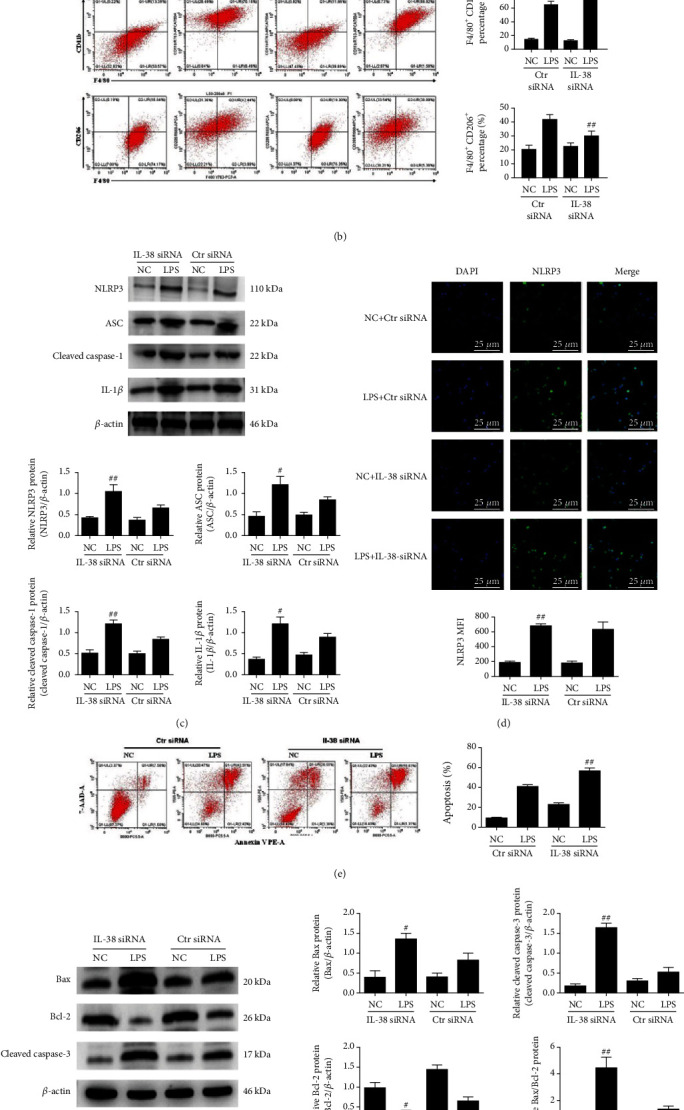
IL-38 downregulation impacts polarization and inflammasome activation and apoptosis in LPS-treated macrophages. (a) Representative western blot image (left panels) and quantification (right panels) of indicated proteins in macrophages treated as indicated. *β*-Actin protein levels were used for normalization. (b) Representative flow cytometry plots of macrophages treated with PBS or LPS (1 *μ*g/mL; 24 h) alone or with anti-IL-38 siRNA in order to quantify populations with the M1 phenotype (F4/80^+^ CD11b^+^) or M2 phenotype (F4/80^+^ CD206^+^). (c) Representative western blot image (upper panels) and quantification (lower panels) of indicated proteins in macrophages treated as indicated. *β*-Actin protein levels were used for normalization. (d) Representative immunofluorescence images of NLRP3 (green) of macrophages treated as indicated. Nuclei were stained with DAPI (blue). Magnification, ×600. Scale bar = 25 *μ*m. (e, f) Representative flow cytometry plots of macrophages treated with PBS or LPS (1 *μ*g/mL; 24 h) alone or with recombinant IL-38 (200 ng/mL; 24 h) in order to evaluate apoptosis (left panel). Percentages of apoptotic (Annexin V+) macrophages treated as indicated (right panel). Representative western blot image (left panels) and quantification (right panels) of the indicated proteins in macrophages stimulated with LPS (1 *μ*g/mL; 24 h). *β*-Actin protein was used for normalization. ^∗^*P* < 0.05, vs. the Ctr siRNA group. ^#^*P* < 0.05 and ^##^*P* < 0.01, vs. the LPS+Ctr siRNA group. Results reflect three independent experiments.

Our results above indicated that IL-38 can influence the inflammatory phenotype and apoptosis of LPS-treated peritoneal mouse macrophages. To confirm this, we knocked down IL-38 in microphage culture using siRNA (Figure 5(a)). Treating these macrophages with LPS increased the shift from anti-inflammatory M2 to proinflammatory M1 phenotype (Figure 5(b)). Knockdown also upregulated NLRP3, ASC, IL-1*β*, and cleaved-caspase 1, corresponding to reduced activation of the NLRP3 inflammasome (Figures 5(c) and 5(d)). At the same time, knockdown increased the number of apoptotic macrophages as well as levels of Bax and cleaved-caspase 3 while decreasing expressions of Bcl-2 (Figures 5(e) and 5(f)). In other words, IL-38 knockdown reinforced the effects of LPS treatment, supporting the idea that IL-38 can help alleviate LPS-induced inflammation and apoptosis.

### 3.6. IL-38 Influences Mortality of Septic Mice

**Figure 6:**
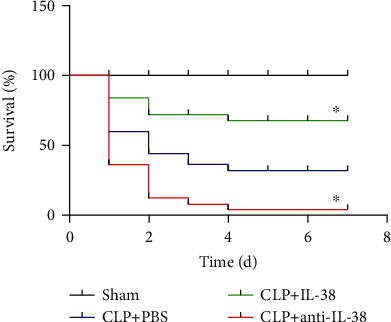
Effects of IL-38 stimulation or blockade on 7-day survival of septic mice. Kaplan Meier survival plot of mice treated as indicated. Septic mice were given PBS (blue line) or IL-38 (1 *μ*g per mouse), then subjected 2 h later to cecal ligation and puncture (CLP) (green line). Other animals were subjected to CLP, then immediately injected intraperitoneally with anti-IL-38 antibody (50 *μ*g per animal), followed 24 h later by a booster dose of 50 *μ*g (red line). ^∗^*P* < 0.05 vs. the CLP+PBS group. Results are shown for 25 mice per group.

To further explore the role of IL-38 in vivo, we studied the impacts of IL-38 on CLP mice. In our previous study, we reported that IL-38 can exert therapeutic benefits in the CLP mouse model of sepsis, as long as the cytokine is delivered at an early stage [20]. Here, we validated our previous result and further observed that inactivating IL-38 using antibody reduced the survival of such mice (Figure 6).

### 3.7. IL-38 Treatment Alleviates Inflammation and Organ Injury in Septic Mice

**Figure 7:**
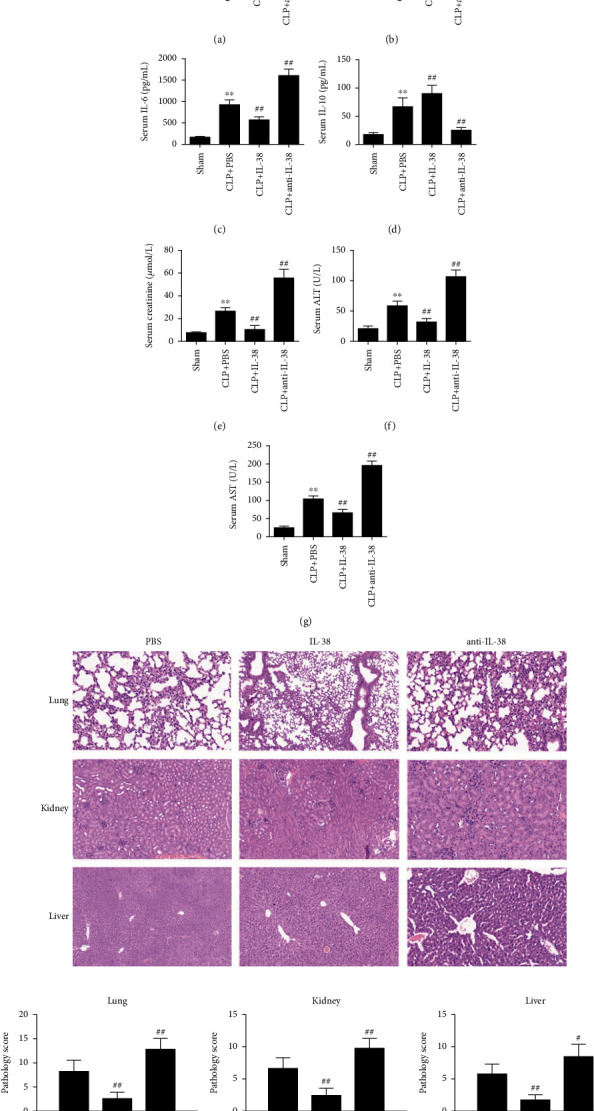
Effects of IL-38 on CLP-induced inflammation and markers of vital organ injury in septic mice. (a–g) Serum levels of indicated inflammatory biomarkers in CLP mice treated with IL-38 or anti-IL-38 antibody. ^∗∗^*P* < 0.01 vs. the sham group; ^##^*P* < 0.01 vs. CLP control. (h) Mice that underwent CLP were treated with IL-38 or PBS. Mice were sacrificed 24 h after CLP. H&E staining and histological scores of the lungs, kidneys, and liver. ^##^*P* < 0.01 vs. the PBS group. Results are shown for six mice per group. ^#^*P* < 0.05 and ^##^*P* < 0.01 vs. PBS control.

To explore how IL-38 might protect against sepsis-associated injury, we quantified IL-38 levels in serum from mice with CLP-induced sepsis. Pretreatment with IL-38 before CLP reduced the CLP-induced increase in serum concentrations of the proinflammatory cytokines TNF-*α*, IL-1*β*, and IL-6 (Figures 7(a)–7(c)), while it enhanced systemic levels of anti-inflammatory IL-10 (Figure 7(d)). IL-38-treated mice also showed lower levels of creatinine, ALT, and AST (Figure 7(e)–7(g)) and lower pathology scores of the lungs, kidneys, and liver (Figure 7(h)), indicating a reduction in tissue damage. Conversely, treatment with anti-IL-38 antibody exacerbated the ability of CLP to upregulate TNF-*α*, IL-1*β*, IL-6, creatinine, ALT, AST (Figure 7(e)–7(g)), and pathology scores (Figure 7(h)), while it partially reversed the ability of CLP to upregulate IL-10 (Figure 7(d)).

## 4. Discussion

Sepsis is a heterogeneous syndrome characterized by systemic inflammation, dysregulated host immune response, and multisystem organ injury [2, 3]. Immune dysfunction lies at the core of sepsis pathogenesis, with macrophages playing key roles through their phagocytosis, production of inflammatory molecules, polarization phenotype, and bactericidal activity [23]. Accordingly, there has been increasing interest in the modulation of macrophage immune activities for sepsis treatment. In the present study, we identified the cytokine IL-38 as a potential treatment against sepsis that acts by altering macrophage-mediated host immune responses.

IL-38 is abundant in various tissues [15] and is implicated in the development of numerous inflammatory disorders, including rheumatoid arthritis, psoriatic arthritis, systemic lupus erythematosus, and asthma [16]. Serum levels of IL-38 are dramatically elevated in septic patients and negatively correlate with proinflammatory biomarkers and blood leukocytes, suggesting that IL-38 is a novel biomarker for identifying sepsis [24]. We found that LPS upregulates IL-38 in CD4^+^CD25^+^ regulatory T cells in septic mice, causing the improvement of host immune function and prognosis in the context of sepsis [20]. The present experiments add macrophages to the list of immune cells that contribute to IL-38 expression in sepsis.

Excessive apoptosis in macrophages may contribute to sepsis-induced immunosuppression and organ injury. IL-38 is released from apoptotic or necrotic macrophages, and it suppresses Th17 maturation [17]. In the current study, we observed the proapoptotic effects of LPS stimulation on macrophages *in vitro*, in a dose- and time-dependent manner. Our data identified IL-38 as a potent inhibitor of sepsis-induced macrophage apoptosis, evidenced by downregulation of apoptosis, Bax, and cleaved caspase 3, with upregulation of Bcl-2 level, leading to an increase in the Bax/Bcl-2 ratio. Proapoptotic Bax and antiapoptotic Bcl-2 act as the core regulators of the intrinsic pathway of apoptosis. The downstream signal caspase 3 is cleaved to be the active form (cleaved caspase 3) and is vital in promoting apoptosis. The role of IL-38 in reducing macrophage apoptosis was confirmed by silencing IL-38. Our data suggest that IL-38 may help limit sepsis-induced immune dysfunction.

IL-38 was previously reported to inhibit the levels of the proinflammatory cytokines IL-22 and IL-17 in peripheral mononuclear cells stimulated by *Candida albicans* infection [15]. Consistently, we found that IL-38 promotes anti-inflammatory M2 macrophage differentiation and prevents proinflammatory M1 differentiation after exposure to LPS. We also observed that administering IL-38 to mice with CLP-induced sepsis lowered the circulating concentrations of the proinflammatory cytokines IL-1*β*, IL-6, and TNF-*α*, while elevating systemic levels of the anti-inflammatory cytokine IL-10. Conversely, IL-38 blockade dramatically aggravated the inflammatory responses and increased differentiation from M2 to M1 macrophages.

Our experiments suggest that LPS and IL-38 exert at least some of their effects in sepsis by influencing the NLRP3 inflammasome. The NLRP3 inflammasome is a multiprotein complex containing NLRP3, its adaptor ASC, its effector caspase 1, and IL-1*β*. In the presence of pathogen-associated molecular patterns or damage-associated molecular patterns, NLRP3 is recruited to cleave precursor pro-IL-1*β* into the proinflammatory form IL-1*β*. The NLRP3 inflammasome has emerged as a potent proinflammatory mediator of the innate immune system [24] and a driver of various inflammatory disorders such as sepsis, inflammatory bowel disease, and atherosclerosis [25–27]. Recently, Luo et al. reported that IL-38 acted as a protective factor in a temporomandibular joint cartilage. In fact, IL-38 could suppress chondrocyte inflammation and protect the cartilage in a manner associated with the NLRP3 pathway [28]. In the present study, we show that NLRP3 is activated in LPS-stimulated macrophages, consistent with previous findings. This process presumably contributes to detrimental inflammatory responses and inflammation-associated programmed cell death, subsequently leading to multiple organ damage [24–26]. Importantly, we found that IL-38 can dramatically inhibit the NLRP3/IL-1*β* signaling pathway by downregulating IL-1*β*, caspase 1, ASC, and NLRP3. We confirmed these effects of IL-38 in knockdown experiments. Our results suggest that IL-38 exhibits potent anti-inflammatory activities partially via suppression of NLRP3 inflammasome activation.

Our studies here on the inflammasome were conducted *in vitro*. Future studies *in vivo* should explore how IL-38 affects NLRP3 inflammasome and apoptosis *in vivo*. Here, we focused on the therapeutic impacts of IL-38 during the early stage of sepsis. The role of IL-38 in later phases of sepsis remains to be explored.

It is known that excessive inflammation and cytokine storms are closely associated with severity of organ dysfunction during sepsis [5]. In our study, IL-38 treatment lowered systemic concentrations of creatine, AST, and ALT in septic mice, implying that it mitigates injury to vital organs. In fact, administration of IL-38 improved the survival of septic mice, while antibody neutralization of IL-38 worsened survival. These *in vivo* studies reinforce the potential of IL-38 as a therapy in sepsis.

## Data Availability

All data in the current study are available from the corresponding authors on reasonable request.
